# Online Anomaly Detection for Smartphone-Based Multivariate Behavioral Time Series Data

**DOI:** 10.3390/s22062110

**Published:** 2022-03-09

**Authors:** Gang Liu, Jukka-Pekka Onnela

**Affiliations:** Department of Biostatistics, Harvard T.H. Chan School of Public Health, Boston, MA 02115, USA; onnela@hsph.harvard.edu

**Keywords:** online learning, anomaly detection, Hotelling’s T-squared test, digital phenotyping

## Abstract

Smartphones can be used to collect granular behavioral data unobtrusively, over long time periods, in real-world settings. To detect aberrant behaviors in large volumes of passively collected smartphone data, we propose an online anomaly detection method using Hotelling’s T-squared test. The test statistic in our method was a weighted average, with more weight on the between-individual component when the amount of data available for the individual was limited and more weight on the within-individual component when the data were adequate. The algorithm took only an O(1) runtime in each update, and the required memory usage was fixed after a pre-specified number of updates. The performance of the proposed method, in terms of accuracy, sensitivity, and specificity, was consistently better than or equal to the offline method that it was built upon, depending on the sample size of the individual data. Future applications of our method include early detection of surgical complications during recovery and the possible prevention of the relapse of patients with serious mental illness.

## 1. Introduction

Digital phenotyping has been defined as “the moment-by-moment quantification of the individual-level human phenotype in situ using data from personal digital devices,” in particular smartphones [1]. Passively collected smartphone behavioral data [2] consist of data from sensors, such as the built-in Global Positioning System (GPS) and accelerometer, as well as phone usage data, such as communication logs and screen activity logs. Anomalies in such multivariate time series (MTS) have been shown to be predictive of relapse for patients with schizophrenia [3,4] and depressive symptoms for women at risk of perinatal depression [5]. Barnett et al. [3] proposed an unsupervised semi-parametric anomaly detection method that is robust against mis-specification of the distribution of a time series. The method was applied to a passively collected smartphone behavioral dataset to detect an escalation of symptoms or signs of a potential relapse. The method decomposes the observed MTS into a general trend, a periodic component, and an error component for each dimension. The error components are then used to build Hotelling’s T-squared test statistic and identify anomalies. Henson et al. [6] applied the method to predict relapse in schizophrenia and achieved 89% sensitivity and 75% specificity in a cohort of 126 participants followed for 3–12 mo.

There are two main limitations to the method described by Barnett et al. [3]. First, the offline algorithm is used mainly to identify anomalous behaviors in a one-time retrospective analysis, where computational performance is not critical. If, however, the goal is to carry out anomaly detection as data are being collected over time (and possibly even in real time) rather than at the end of a data collection period, the method needs to scale to large cohorts of individuals followed for months or years. Second, the offline method uses only within-individual comparisons to overcome the heterogeneity of the data and requires at least two weeks of data to establish the individual baseline for comparisons. This approach is not ideal for anomaly detection in many health-related settings, from surgery to rehabilitation, in which the time period immediately following patient discharge is high risk for adverse events. Forster et al. [7] found that nearly 20% of patients experience adverse events within three weeks of discharge. Meta-analyses of suicide rates after discharge from psychiatric facilities suggest that these rates remain high for several years, but are particularly high in the first few weeks and months post-discharge [8,9,10]. Given that this time period is most likely to incur anomalies, methods should perform well during this period. Last but not least, since the primary goal of smartphone-based digital phenotyping is to passively monitor health, there are no labels available from surveys or other sources of active data.

Recent research on unsupervised online anomaly detection mainly focuses on improving accuracy using a customized neural network or a clustering method designed for a specific scenario. Hsieh et al. [11] proposed an algorithm using a long short-term memory (LSTM)-based autoencoder for smart manufacturing; Aminanto et al. [12] used the isolation forest method to solve the threat-alert fatigue problem; Yu et al. [13] proposed an algorithm called DDCOL, a density-based clustering method to detect anomalies in various key performance indicators for IT companies; Karaahmetoglu et al. [14] combined LSTM networks with a support vector data descriptor to process irregularly sampled sequences; Hwang et al. [15] presented an anomaly traffic detection mechanism, D-PACK, which consists of a convolutional neural network and an autoencoder for auto-profiling the traffic patterns and filtering abnormal traffic. Jones et al. [16] applied an adaptive resonance theory artificial neural network to identify cyberattacks on Internet-connected photovoltaic system inverters; Scaranti et al. [17] developed an intrusion detection system based on online clustering to detect attacks in an evolving network.

Our goal was to develop a lightweight, unsupervised online anomaly detection method for passively collected smartphone behavioral multivariate time series data. We specifically looked for a method that had low computational complexity and the ability to detect anomalies after minimal training. To realize these goals, we took a different approach from the research summarized above, and instead, we revisited the method of Barnett et al., which has already proven effective in detecting anomalies in smartphone behavioral data [3,6]. We review the method of Barnett et al. in Section 2.1, describe our new method in Section 2.2, illustrate our method using simulated and real data in Section 3, and discuss our method in Section 4.

## 2. Materials and Methods

### 2.1. Offline Anomaly Detection Method

In their offline method, Barnett et al. [3] first defined the expected behavior for each individual by decomposing the observed MTS of *p* features into an overall trend, a weekly component, and an error component for each dimension. For a given individual, let $m_{i}$ be the number of days of follow-up where feature *i* is observed. Let $y_{ij}=\mu_{ij}+s_{ij}+\epsilon_{ij}$ be the value of the *i*th feature on day *j* of follow-up, where $\epsilon_{ij}$ is the error component and $\mu_{ij}$ is the trend component estimated from a weighted average of the previous observed feature values $y_{i,j-1},y_{i,j-2},\ldots$, with more weight given to observations closer in time. These weights are specified as a *t*-distribution with two degrees of freedom and scaling parameter $10/\max m_{i}$. The weekly component $s_{ij}$ is estimated to minimize the square error under the restriction $s_{ij}=s_{i,j-7}$. After estimating the decomposition of the time series as ${\hat{\mu }}_{ij},{\hat{s}}_{ij},{\hat{\epsilon }}_{ij}$, the authors transformed the errors ${\hat{\epsilon }}_{ij}$ non-parametrically into *Z*-scores by sorting the errors by rank across all days of follow-up for that feature, followed by a standard normal transformation using the probability integral transform. Mathematically, the transformed error ${\tilde{\epsilon }}_{ij}$ can be expressed as ${\tilde{\epsilon }}_{ij}=\Phi^{-1}\left(\frac{\mathrm{rank}({\hat{\epsilon }}_{ij})}{m_{i}+1}\right)$, with ${\tilde{\epsilon }}_{j}={\left[{\tilde{\epsilon }}_{1j},\ldots ,{\tilde{\epsilon }}_{pj}\right]}^{T}$ denoting the vector of transformed errors on day *j* and ${\tilde{\epsilon }}_{k}^{*}={\left[{\tilde{\epsilon }}_{k1},\ldots ,{\tilde{\epsilon }}_{k,m_{i}}\right]}^{T}$ denoting the vector of transformed errors of feature *k*. The covariance between the transformed errors of feature *i* and feature *k* is defined as $\Sigma_{ik}=\mathrm{cov}\left({\tilde{\epsilon }}_{i}^{*},{\tilde{\epsilon }}_{k}^{*}\right)$, which is estimated empirically across all days where both are observed. Hotelling’s T-squared test statistic was constructed as $Q_{j}={\tilde{\epsilon }}_{j}^{T}\Sigma^{-1}{\tilde{\epsilon }}_{j}$, where $Q_{j}$$\overset{p}{\to }$$\chi_{p}^{2}$ under the null hypothesis that the observation is not anomalous on day *j*. To correct for multiple comparisons, the method bootstraps the error components of the time series assuming stationarity to generate the null distribution for the largest test statistic across all days of follow-up, and the α-quantile of the bootstrapped values provides the threshold for significance at the α significance level.

The offline method was designed for retrospective analyses in studies with no intervention component, where detecting anomalies at the end of data collection is sufficient and computational performance is not critical. Notably, there are four steps to the offline algorithm with linear or super-linear computational complexity: (1) calculating the general trend using the weighted average of all historical observations, (2) estimating the periodic term $s_{ij}$ through linear regressions, (3) sorting the errors of each feature, and (4) computing the empirical covariance matrix of the transformed errors. The value of anomaly detection lies in the ability to detect anomalies and act on them in as close to real time as possible. Although the offline method described above could be applied repeatedly, this would be computationally very expensive. The method also needs at least two weeks of data to establish baselines for a given individual; yet in practice, these first two weeks of collected data may be the most likely to have anomalies that need to be acted upon, for example if they coincide with patient discharge from a facility, as discussed above. These considerations motivated our online anomaly detection method, presented in the next section.

### 2.2. Online Anomaly Detection Method

In this section, we address the different components of our method separately.

#### 2.2.1. Updating the General Trend and Periodic Terms

Estimation of both periodic and non-periodic trends requires assigning weights to past observations. Even though a *t*-distribution with two degrees of freedom of the offline method has thick tails, the weights for observations far away from the current observation become negligible when $m_{i}$ is large. Instead of using all historical data to compute the average, we propose to use a subset of *K* most recent observations to reduce both computational time and memory use. The periodic term in the original method was estimated through linear regression, where the effect size of each day of the week is expressed as the mean observed residual on that day of the week. Here, we used sample means instead of linear regressions to estimate the periodic terms. The new estimates are identical to those from linear regression, but given that we used the classic online approach [18] to calculate the mean, the computational complexity was O(1), and it used less memory, as only a running sum and the number of observations need to be stored.

#### 2.2.2. Sorting the Errors

Our proposed online algorithm requires the ranks of the errors within each feature in each update. The values of the errors are not fixed over time, but change periodically, which makes the online sorting procedure a non-trivial problem. For example, if a data point on the Wednesday of the third week is observed, then the periodic term for Wednesdays is updated and the current error is computed. Assuming the new estimate of the periodic term is larger than the previous estimate by δ, the values of the errors for the first and second Wednesday should both be decreased by δ in this update, given the decomposition $y_{ij}=\mu_{ij}+s_{ij}+\epsilon_{ij}$. Furthermore, given that $\mu_{ij}$ is fixed once estimated, the rank of the current error cannot be obtained by locating the index of the previously sorted error. Rather than sorting the data from scratch in each update as in the offline method, we took advantage of the trackable changes in the errors estimated by sample means and propose a binning method that obtains approximate ranks of both the current error and all previous errors by examining the quantiles of the empirical distribution of all errors.

We illustrate this idea assuming a weekly period. For each feature, we initialized a histogram for the errors for each day of the week with *H* bins using the first *M* observations. The bin width *w* for each feature was determined by the corresponding maximum and minimum values $w=\frac{R(\max\{S\}-\min\{S\})}{H}$, where $S=\left\{y_{k}|k=1,2,\ldots ,M\right\}$, $y_{k}$ is the feature value on day *k* and R(>1) is a hyperparameter that controls the range that the histogram can cover on the flanks of the observed range for unobserved future values. The locations of the bins for each feature are lined up across the seven days of the week. When we applied the method and observed a new value $y_{n}$ on day *j* of the week after *M* days, we first updated the estimate of $s_{j}$ using the sample mean, then calculated the difference between the new and old estimates $\delta_{j}=s_{j,n}-s_{j,n-1}$ and the new error $\epsilon_{n}=y_{n}-\mu_{n}-s_{j,n}$. If the difference $\delta_{j}$ was positive, the values of all previous errors on day *j* of the week decreased by $|\delta_{j}|$, which caused the corresponding histogram of the errors to shift $\left\lfloor |\delta_{j}|/w_{j}\right\rceil$ bins to the left, where ⌊⌉ denotes rounding to the nearest integer. If the difference was negative, we shifted the histogram to the right. We then located the bin of the new error $\epsilon_{n}$ and updated the histogram again by adding one to the count of observations in that bin. Finally, we aggregated all histograms for the seven days of the week and summed up the count in each bin to obtain a final histogram of all errors. We located the percentile of the new error $\epsilon_{n}$ by dividing the sum of counts, starting from the leftmost bin and proceeding to the right, until we obtained a bin for which $\epsilon_{n}$ fell within the total sum. We converted the percentile to a normal random variable using the inverse standard normal cumulative distribution function. The number of bins *H* determines the precision of this method; empirically, H=100 appeared to achieve good performance.

#### 2.2.3. Updating the Covariance Matrix

The classic online approach for updating the covariance matrix is to decompose the new covariance matrix as a weighted sum of the old covariance matrix and an outer product of the new error vector ${\tilde{\epsilon }}_{n}$, which can be expressed as ${\hat{\Sigma }}_{n}=\frac{n-1}{n}{\hat{\Sigma }}_{n-1}+\frac{1}{n}{\tilde{\epsilon }}_{n}{\tilde{\epsilon }}_{n}^{T}$, where $\Sigma_{j}$ denotes the covariance matrix after the *j*th update. However, in our setting, because the values of all previous $\tilde{\epsilon }$ change when a new data point is observed, we could not apply the method directly to our problem. Note that each element in $\tilde{\epsilon }$ is obtained by the standard normal transformation; thus, $\tilde{\epsilon }$ is a multivariate normal random vector with a variance of each dimension 1 by the Cramér–Wold device, and the covariance matrix of $\tilde{\epsilon }$ is essentially a correlation matrix. Motivated by this observation, we propose an approximation whereby we re-scaled the covariance matrix obtained by the classic approach to a correlation matrix after each update. This was accomplished by multiplying the inverse diagonal matrix filled by the standard deviation of each dimension in the front and at the end of the covariance matrix, which can be expressed as $\mathrm{corr}=D^{-1}\Sigma D^{-1}$, where $D=\sqrt{\mathrm{diag}(\Sigma )}$.

Thus far, every step in the offline method was modified to an online algorithm, summarized as follows. Given a new observation, we decomposed it as described above and computed the test statistic using the histograms and covariance matrix from the previous update. If the corresponding *p*-value was smaller than the threshold, we sampled a dummy variable *I* from Bernoulli(*p*). If I=0, we classified the current observation as an anomaly; otherwise, we considered it normal and updated the histogram and covariance matrix.

#### 2.2.4. Incorporating the between-Individual Comparison

Under the assumption of weekly periodicity, the method proposed by Barnett et al. [3] uses only within-individual comparisons, and thus requires at least two weeks of data from each individual to detect anomalies. For clinical applications, anomaly detection is most helpful during periods of time when anomalies are most likely to occur, for example following some intervention, such as surgery or rehabilitation. The risk of adverse events or relapse is usually highest soon after the intervention, which is also the time period when there may be little to no data collected from the individual. To address this limitation, our method borrows information from other individuals so that it can identify anomalies starting from the first day of follow-up. Though passive behavioral data exhibit a high level of heterogeneity among individuals, a cohort-level baseline is still an acceptable benchmark to start with if little to no information about a given individual is available. Similar to the method described in Section 2.2, we constructed a cohort-level histogram of the original feature values for each feature and each day of the week, and the percentile of each observation in the cohort was used to derive the chi-squared test statistic. The histograms were updated by simply adding the count of new observations in each bin; no shifting or other manipulations were needed. Let $Q_{b}$ denote the test statistic derived from cohort-level (between-individual) percentiles and $Q_{w}$ denote the test statistic derived from within-individual percentiles. We propose a weighted average of the two, namely $Q=wQ_{b}+\left(1-w\right)Q_{w}$, as the final test statistic, where $Q\overset{p}{\to }\chi_{p}^{2}$ since $Q_{b}$ is asymptotically independent of $Q_{w}$ in the number of individuals. The value of *w* should be one in the first two weeks, and it should vanish gradually as more data become available for the individual. In addition, we suggest the use of a dynamic significance level to identify anomalies in practice. For example, we could set α=0.1 for the first month and decrease it gradually to 0.05 over time. The trajectories of both *w* and α should be tailored for specific settings and should depend on the relative likelihood of early vs. late anomalies (e.g., adverse events, relapse).

#### 2.2.5. Software Implementation

Our group developed the open-source Beiwe data collection platform for smartphone-based digital phenotyping, with continuous development and use since 2013 [19]. We also recently released Forest, an open-source Python data analysis library for Beiwe data. Forest can be run independently of Beiwe, but the primary use case is for the two tools to be fully integrated directly on the Amazon Web Services (AWS) back-end. Cloud-based data analysis obviates the need to move large volumes of data and allows the implementation of the preferred big data computing paradigm where computation is taken to data rather than vice versa. It also makes the system more readily compliant with regional data privacy regulations, such as the General Data Protection Regulation 2016/679 (GDPR) in the European Union law that protects data and privacy in the European Union and the European Economic Area [20].

We implemented the proposed online anomaly detection method as a module within Forest. Thus, in addition to running the method using existing data, interested readers can collect their own data using Beiwe and then run the online anomaly detection algorithm as part of Forest on a daily basis. Results can be stored in a database in the AWS back-end, and the open-source implementation provides an API for using Tableau or similar software to visualize the results. The Forest module that implements the method as described in this paper is called Banyan [21]. It consists of eight user-configurable parameters, including the period of the data, the number of bins in the histogram, and the significance level.

## 3. Results

### 3.1. Simulation with Synthetic Data

**Figure 1:**
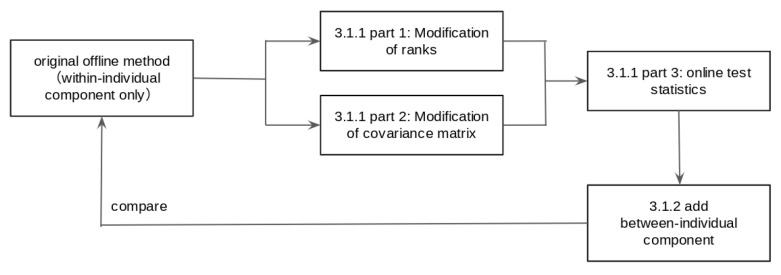
A flowchart of the simulation study described in Section 3.1.

The test statistic in our online method consists of a within-individual component as the counterpart of the test statistic in the Barnett et al. method [3] and a between-individual component. We studied two important aspects of the method. First, we compared the within-individual component of the online test statistic with the offline test statistic. Second, we compared the performance of the online method with the weighted test statistic and the offline method. The logic is displayed in the flowchart in Figure 1. Our findings showed that the value of the within-individual component of the test statistic approximates the offline test statistic, but is faster to compute. Our proposed method of using the weighted average of both components worked well in the first two weeks of data collection, and its performance in terms of sensitivity and specificity converged to the offline method when the follow-up period was long enough.

#### 3.1.1. Comparison of the within-Individual Component of the Online Test Statistic and the Offline Test Statistic

The within-individual component was derived using a two-step online algorithm, where we first obtained the rank-based transformed errors from the observed features and then updated the covariance matrix using these errors. We examined (1) the difference between the ranks given the same observed features, (2) the difference between the covariance matrices given the same transformed errors, and (3) the difference between the test statistics given the same observed features for the two methods. We generated the observed features using the decomposition $y_{ij}=\mu_{ij}+s_{ij}+\epsilon_{ij}$, where $\mu_{ij}=0,s_{ij}∼N\left(0,2\right),s_{ij}=s_{ij+7}$. The error term $\epsilon_{j}=\left[\epsilon_{1j},\ldots ,\epsilon_{pj}\right]$ was generated in three different ways: (1) a standard multivariate normal distribution, (2) *p* independent gamma distributions with α=2, β=0.5, and (3) a multivariate normal distribution with a correlation of 0.7 between any two features. The number of features was set to 20, 40, and 80, and the number of bins in the histogram was set to 50, 100, and 500. The data generation procedure was repeated ten times, and the results shown below are the averages of the replicates.

##### Comparison of Ranks

**Figure 2:**
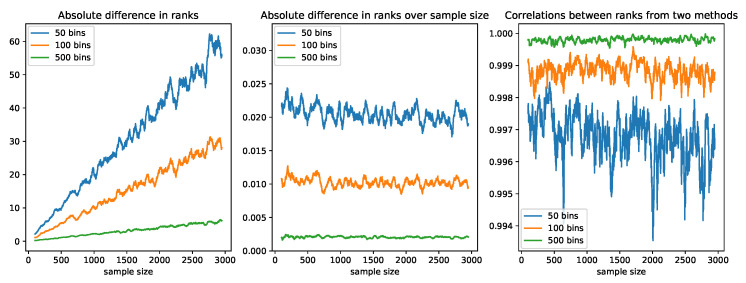
A comparison of the ranks obtained by sorting from scratch and using the online histogram method. The left panel shows the average absolute difference between the two sets of ranks using the most recent 50 observations in each update averaged over five replications. The middle panel shows the average absolute difference over the sample size, and the right panel shows the Spearman correlation in the same setting.

In each scenario, the ranks of the errors from our online algorithm were obtained by updating the histograms as described above, whereas the ranks from the offline method were derived by sorting errors from scratch in each update. We initialized the histograms using the first 100 observations and compared the ranks of the two methods starting from Observation 101. We computed the average absolute difference, the average absolute difference divided by the sample size, and the Spearman correlation between the two sets of ranks using the most recent 50 observations in each update. Since the ranking procedure happens within each feature, we only studied how the number of bins affected the correlation using independent normal errors. As shown in Figure 2, the absolute difference between the two sets of ranks increased as the sample size increased. This happened because ranks that were close to one another ended up in the same bin. However, when we divided the absolute difference by the sample size, we found that the ratio converged to the reciprocal of the number of bins. This means the expected deviation in ranks was 1/H of the sample size. The Spearman correlation was consistently above 99.5% in all three scenarios, and the correlation was greater for more granular histograms (those with a greater value of *H*).

##### Comparison between Covariance Matrices

**Figure 3:**
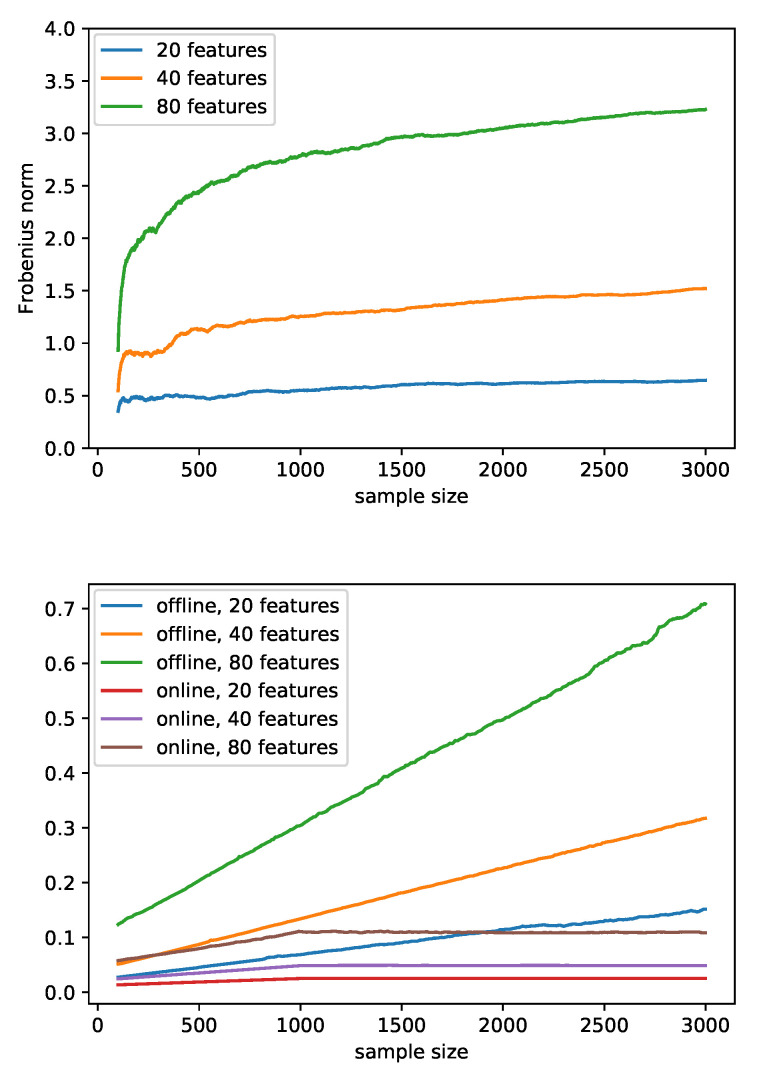
The upper panel shows the Frobenius norm of the difference between the covariance matrices obtained from the empirical estimation and our proposed online algorithm in each update averaged over five replications. The transformed errors were taken from the offline method and used in both methods to calculate the covariance matrices. The lower panel is a comparison of the runtime between the offline method and our proposed online method as the sample size increases from 100 to 3000. The runtime in each update was measured in seconds with an Intel^®^ Xeon^®^ CPU E5-2697 v3 @ 2.60GHz CPU. Since the difference in time caused by different numbers of bins and different distributions of errors are too small to be seen on the graph, an average line is used to represent all the scenarios given the method and the number of features.

As the covariance matrix depends on the transformed errors and those errors are different between our online method and the offline method, for the purposes of this simulation, we used the errors from the offline method to examine the performance of the modified covariance updating algorithm for both methods. In our method, the covariance matrix was updated as described in Section 2.2.3, while in the offline method, it was estimated empirically from scratch in each update. We investigated the Frobenius norm of the difference of the two matrices using simulated data with different numbers of features. As presented in the upper panel in Figure 3, the Frobenius norm of the difference was small, but grew with the number of updates. After sufficiently many updates, the norm converged. The norm of the difference was larger when the number of features was larger due to the higher dimensionality of the difference matrix.

##### Comparison of the Test Statistics

We evaluated our two-step algorithm to compute the within-individual test statistic and focused on the distribution of the test statistic. The same features were used for both algorithms, and the corresponding computation time and test statistics were compared in various scenarios. The lower panel in Figure 3 shows that the runtime of each update increased linearly as the sample size grew for the offline method and was greater than that of our proposed method even when the sample size was small. The runtime of our method also increased linearly, but more slowly in the first 900 updates; it then became constant because we chose K=1000 (the size of the subset in Section 2.2.1) as the maximal number of historical values in memory to determine the general trend in this example. Additionally, the runtime was positively associated with the number of features. Since the difference in runtime caused by different numbers of bins and different error distributions are too small to be seen on the graph, an average line is used to represent each scenario.

**Figure 4:**
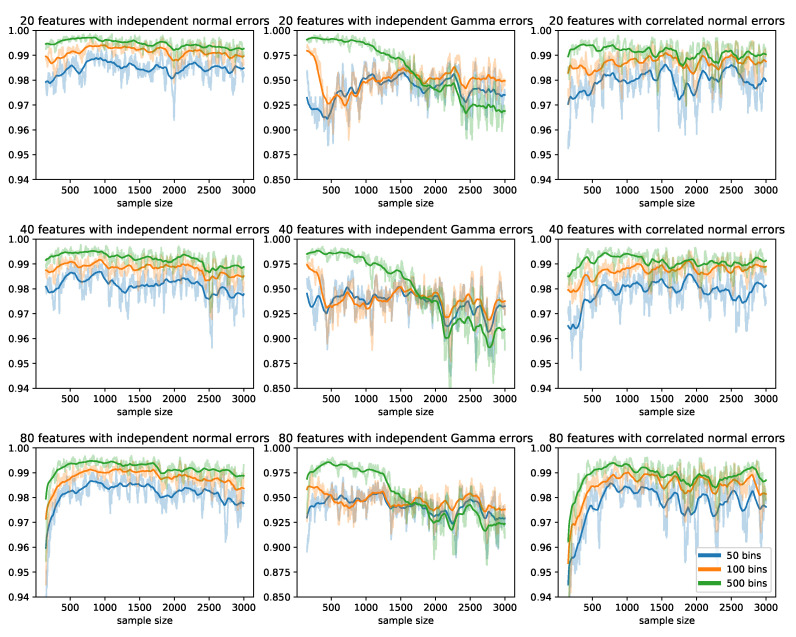
Spearman correlations between the test statistics obtained from the offline method and our proposed online method using the most recent 50 updates in each update. Each row represents a different number of features; each column represents a different distribution of the error terms; each color represents a different number of bins in the histogram method.

Figure 4 shows the Spearman correlation between the test statistics from the offline method and our proposed method using the most recent 50 updates in each update. In the scenarios where the errors were generated from independent or correlated multivariate normal distributions, the correlations were consistently higher than 0.95 after the first 200 updates; increasing the number of bins resulted in higher correlations and lower variances. In scenarios where the errors were generated from independent Gamma distributions, the correlations between the two sets of test statistics fell to 0.9 and stabilized after 2000 updates.

**Figure 5:**
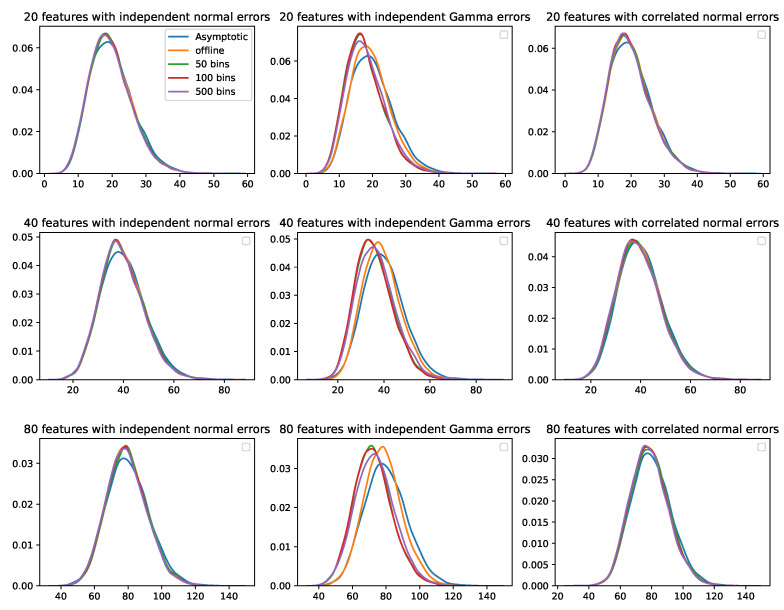
Empirical distributions of the test statistics obtained from the offline method and our proposed online method, compared to a standard $\chi^{2}$ distribution with a degree of freedom equal to the number of features. Each row represents a different number of features; each column represents a different distribution of error terms; each color represents a different method.

Figure 5 depicts the distributions of the test statistics from various methods compared to a standard $\chi^{2}$ distribution with a degree of freedom specified as the number of features. The density plots of the test statistics from the offline method and our method coincided when the errors were normally distributed. However, when the errors followed a Gamma distribution, the mean of the test statistics from our method was smaller than the mean from the offline method and the mean of the asymptotic distribution. The absolute value of the difference was positively associated with the number of features.

#### 3.1.2. Comparison of the Performance of the Online Method with the Weighted Test Statistic and the Offline Method

To simulate anomalies in the features, we generated an MTS using the sine function with different scale ($a_{j}$) and phase ($b_{j}$) parameters, namely $T\left(t_{ij}\right)=a_{j}\sin\left(\frac{t_{ij}}{c}+b_{j}\right)$, where *T* is an intermediate variable used later to generate the observed features, *i* denotes the *i*th observation, *j* denotes the *j*th feature, and *c* is a parameter that fixes the periodicity of the function to seven days. Next, we let $y_{i1}=T\left(t_{i1}\right)$ and $y_{ij}=p_{1}T\left(t_{i,j-1}\right)+p_{2}T\left(t_{ij}\right)$ with j>1 and $p_{1}+p_{2}=1$ to induce correlations in the features. Gaussian noise (with zero mean and unit standard deviation) was added to each feature of the original MTS to increase the difficulty of detecting the anomalies and make the data more realistic. To generate artificial anomalies, we randomly selected *m*% of the sample, then for each observation, we again randomly selected [30%,70%] of the features and altered their magnitudes by multiplying them by a uniformly distributed random variable u∼U[0,3]. We replicated the procedure 100 times to generate the observed features for 100 individuals. In each replication, we set the number of features to 10 and the number of observations to 540.

**Figure 6:**
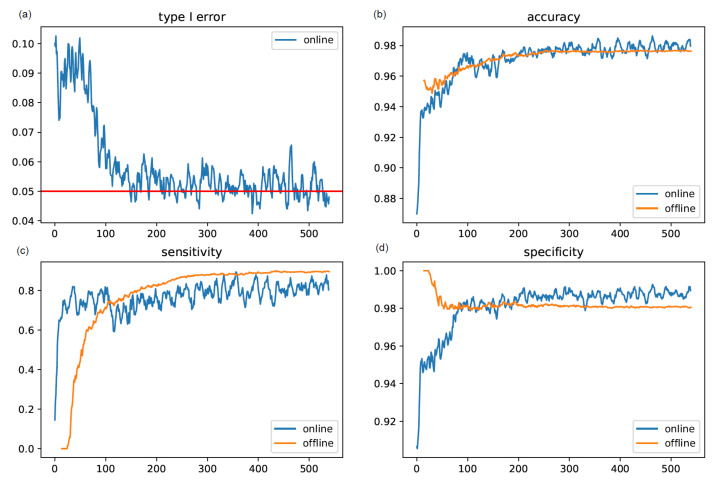
The average false positive rate, accuracy, sensitivity, and specificity of the proposed online method for 540 d across 100 synthetic datasets of 100 individuals. The underlying anomaly rate is 0 for studying the type I error, and the underlying anomaly rate is 0.05 for studying other metrics. The significance level is 0.05.

In one simulation, we set the anomaly rate to zero and the significance level to 0.05 to study the type I error of the online method. Figure 6a shows that the online method had an initially inflated false positive rate, which then decreased to the nominal level after about 100 updates. In another simulation, we set the anomaly rate to 0.05 and the significance level to 0.05. In the online method, the weight of the within-individual component was zero for the first four weeks, increased linearly to one on Day 112, and remained at one afterward. The accuracy, sensitivity, and specificity were calculated after each update and are presented in Figure 6b–d. The online method was able to detect the anomalies in the first 14 d with a corresponding average accuracy, sensitivity, and specificity of 91.2%, 50.4%, and 93.3%, respectively. From Day 15 to Day 112, the online method had a higher sensitivity, but slightly lower specificity. From Day 112 on, the online method only used the within-individual component, and its performance was similar to that of the offline method. Note that, as expected, the runtime of the online method was much faster, as shown in Figure 3.

### 3.2. Simulation with Pseudo-Data

Panda et al. [22] conducted a study to collect raw smartphone accelerometer data continuously for six months from adults who had a cancer diagnosis and were scheduled for surgery between July 2017 and April 2019. The study was designed to discover if smartphones could capture novel postoperative recovery metrics among the patients. Most patients (45, 73%) experienced no clinically significant postoperative events, and those who experienced such an event did not report the exact date of when they started to feel unwell. Since there was no reliable ground-truth available for the timing of these types of anomalies, we instead chose to create artificial anomalies for patients who did not experience any. We constructed a dataset by first calculating the mean of each metric μ for each of the 45 individuals who did not report an anomaly. We then calculated the individual-specific residuals ϵ, a difference vector of the actual observation, and the mean vector for each day. To create a dataset of pseudo-observations with *K* days of follow-up, we bootstrapped the errors *K* times and added them to the mean vector. To create anomalies, in the bootstrapping step, we randomly sampled 5%×K of the residuals and multiplied them by an inflation factor *z*, where z∈{1,2,3,4}. In other words, for each day, the pseudo-observation was generated as μ+zϵ. When z=1, the dataset did not have any anomalies, and we expected our method to recover the nominal false positive rate. We repeated the procedure 50 times and calculated the average accuracy, sensitivity, and specificity every 30 d with various *z* values across the 45 individuals. As shown in Table 1, our online method achieved nominal sensitivity when z=1. When z>1, the sensitivity increased as the sample size increased and then plateaued to a stable level. As expected, the sensitivity was higher for greater values of *z*. The corresponding accuracy is listed in Table 2.

## 4. Discussion and Conclusions

Smartphones are promising tools for detecting behavioral anomalies given the ubiquity of the devices and the feasibility of using them for long-term follow-up, especially if relying on passively collected data. Our online anomaly detection method is simple, and it performed well in the studied setting. We believe that its transparency and interpretability are important strengths in future research and clinical applications.

The proposed online anomaly detection algorithm is a natural extension of the offline method proposed by Barnett et al. [3], and the computational complexity of each update in the method was O(1). The method requires a minimal training dataset and is able detect anomalies starting from the first day, which is particularly important in health studies. In addition, our method can leverage information from other individuals, which is another improvement over the offline method. The performance of the two methods is similar in terms of accuracy, sensitivity, and specificity when the number of observations for each individual is sufficiently large. The online method has also been implemented as a Python package in the Forest library [19].

The proposed method has some limitations. First, the errors derived by the decomposition may not reflect the extent to which the observation is anomalous if the feature is not self-predictive or the pattern is too complex to be approximated by a general trend and a periodic term. Second, the approach requires some expertise from the user to determine a reasonable period for transitioning from cohort-level data to individual-level data. Third, the test statistic is essentially a Mahalanobis distance, which measures the distance between the current observation and the median. It standardizes all features such that they have equal weights in the test statistic. However, some features may be more informative than others, and the current method does not provide a way for determining this possibility. Fourth, the distribution of the test statistic is asymptotically a Chi-squared distribution; however, in practice, the empirical distribution is more concentrated around the mode, and sometimes, the mode can even shift away from the expected value, as we saw with the Gamma distributed errors. This behavior results from approximating the true percentiles using a histogram, a non-continuous grid. Hence, the *p*-value derived from the asymptotic distribution may not be accurate. However, in general, the method is robust against the mis-specification of the distribution since it is rank based. Fifth, the anomalies are determined by a user-specified threshold, the significance level, rather than an estimate of the underlying anomaly rate. Thus, when the true anomaly rate is very small, the method is expected to have a large false positive rate. Finally, in our simulation studies, the estimated mean of the various daily features included data from the anomalous periods. Ideally, when estimating the mean, one should use data from healthy controls only or exclude anomalous time periods from those subjects who experience anomalies. The main challenge with this approach is that it would require knowledge of the existence and timing of the anomalies beforehand, whereas the goal of the method is to detect those anomalies. In practice, the bias in the estimated mean caused by the inclusion of anomalous time periods is very small because anomalies are rare. Furthermore, the inclusion of anomalies in estimating the mean has the effect of reducing the size of the resampled residuals, thus biasing the test towards the null, i.e., making it slightly more conservative.

## Figures and Tables

**Table 1:** The sensitivity of the online method to detect an artificial anomaly at different stages of follow-up among 45 patients over 50 repetitions with an anomaly rate of 0.05. For each day, the data are generated by μ+zϵ, where μ is the mean feature vector, ϵ is the individual-specific residual generated by bootstrapping empirical residuals, and *z* controls the severity of the anomaly.

z\Day	1–30	31–60	61–90	91–120	121–150	151–180
1	0.0566	0.0547	0.0514	0.0501	0.0492	0.0490
2	0.3765	0.4204	0.4143	0.4305	0.4254	0.4352
3	0.4505	0.4949	0.4849	0.4858	0.4915	0.4986
4	0.4558	0.5079	0.5071	0.5216	0.5355	0.5393

**Table 2:** The accuracy of the online method to detect an artificial anomaly, where the accuracy is defined as the rate of a correct classification.

z\Day	1–30	31–60	61–90	91–120	121–150	151–180
1	0.8916	0.8945	0.9016	0.9007	0.8998	0.9023
2	0.9405	0.9446	0.9482	0.9489	0.9485	0.9451
3	0.9482	0.9533	0.9556	0.9577	0.9586	0.9589
4	0.9490	0.9551	0.9586	0.9609	0.9646	0.9648

## Data Availability

Not applicable.
